# Quality and Adoption of COVID-19 Tracing Apps and Recommendations for Development: Systematic Interdisciplinary Review of European Apps

**DOI:** 10.2196/27989

**Published:** 2021-06-02

**Authors:** Leonie Kahnbach, Dirk Lehr, Jessica Brandenburger, Tim Mallwitz, Sophie Jent, Sandy Hannibal, Burkhardt Funk, Monique Janneck

**Affiliations:** 1 Department of Health Psychology and Applied Biological Psychology Leuphana University of Lüneburg Lüneburg Germany; 2 Competencies for Digitally-Enhanced Individualized Practice Project Leuphana University of Lüneburg Lüneburg Germany; 3 Institute for Interactive Systems Technische Hochschule Lübeck Lübeck Germany; 4 Department for Electrical Engineering and Computer Science Technische Hochschule Lübeck Lübeck Germany; 5 Institute of Information Systems Leuphana University of Lüneburg Lüneburg Germany

**Keywords:** COVID-19, contact tracing, app-based tracing, Mobile App Rating Scale, user engagement, human–computer interaction

## Abstract

**Background:**

Simulation study results suggest that COVID-19 contact tracing apps have the potential to achieve pandemic control. Concordantly, high app adoption rates were a stipulated prerequisite for success. Early studies on potential adoption were encouraging. Several factors predicting adoption rates were investigated, especially pertaining to user characteristics. Since then, several countries have released COVID-19 contact tracing apps.

**Objective:**

This study’s primary aim is to investigate the quality characteristics of national European COVID-19 contact tracing apps, thereby shifting attention from user to app characteristics. The secondary aim is to investigate associations between app quality and adoption. Finally, app features contributing to higher app quality were identified.

**Methods:**

Eligible COVID-19 contact tracing apps were those released by national health authorities of European Union member states, former member states, and countries of the European Free Trade Association, all countries with comparable legal standards concerning personal data protection and app use voluntariness. The Mobile App Rating Scale was used to assess app quality. An interdisciplinary team, consisting of two health and two human–computer interaction scientists, independently conducted Mobile App Rating Scale ratings. To investigate associations between app quality and adoption rates and infection rates, Bayesian linear regression analyses were conducted.

**Results:**

We discovered 21 national COVID-19 contact tracing apps, all demonstrating high quality overall and high-level functionality, aesthetics, and information quality. However, the average app adoption rate of 22.9% (SD 12.5%) was below the level recommended by simulation studies. Lower levels of engagement-oriented app design were detected, with substantial variations between apps. By regression analyses, the best-case adoption rate was calculated by assuming apps achieve the highest ratings. The mean best-case adoption rates for engagement and overall app quality were 39.5% and 43.6%, respectively. Higher adoption rates were associated with lower cumulative infection rates. Overall, we identified 5 feature categories (symptom assessment and monitoring, regularly updated information, individualization, tracing, and communication) and 14 individual features that contributed to higher app quality. These 14 features were a symptom checker, a symptom diary, statistics on COVID-19, app use, public health instructions and restrictions, information of burden on health care system, assigning personal data, regional updates, control over tracing activity, contact diary, venue check-in, chats, helplines, and app-sharing capacity.

**Conclusions:**

European national health authorities have generally released high quality COVID-19 contact tracing apps, with regard to functionality, aesthetics, and information quality. However, the app’s engagement-oriented design generally was of lower quality, even though regression analyses results identify engagement as a promising optimization target to increase adoption rates. Associations between higher app adoption and lower infection rates are consistent with simulation study results, albeit acknowledging that app use might be part of a broader set of protective attitudes and behaviors for self and others. Various features were identified that could guide further engagement-enhancing app development.

## Introduction

### COVID-19 Contact Tracing Apps

For the first time, digital technology could play a key role in fighting a global health crisis. Particularly, COVID-19 contact tracing apps have been advocated as a way to achieve pandemic control, avoid or leave lockdowns [1], and return to normalcy [2]. Contact tracing and other nonpharmaceutical measures—like testing, case isolation, quarantining, hygiene, decontamination, and physical distancing—are especially important in situations where vaccinations and effective treatment are not widely available. Even in light of achievements in developing the first available vaccines [3], several countries will have been locked down twice or even three times (eg, Germany and Austria both as of December 2020) before pandemic control is expected to be reached through vaccination dissemination.

The potential advantage of contact tracing apps consists in shared characteristics of digital technology and the SARS-CoV-2 virus: speed and spread. SARS-CoV-2 is being transmitted between people at a high speed. Hinch and colleagues [4] have assumed a generation time for virus transmission of just 6 days and an epidemic doubling time of 3-3.5 days, resulting in a large proportion of the population potentially becoming infected within short periods of time in the absence of any effective intervention. For this reason, modeling study results have continuously suggested that manual contact tracing might be too slow, largely due to personnel limitations, and only feasible in locations with low incidence rates [1,2,5]. Briefly, manual contact tracing is a process by which some index person with confirmed infection reaches out to contact health authority personnel to provide information about other people with whom they have come in contact with and whom they, thereby, might have infected. The health authorities then trace those people to inform them about their possible infection and the necessity that they seek testing or quarantine themselves [5].

Major advantages of digital technology are its large scalability and its potential to speed up the tracing process. Key features of contact tracing apps for COVID-19 consist of informing others immediately of having been in contact with a person who is infected, thus speeding up isolation, testing, and quarantining. Moreover, contact tracing apps with symptom checkers can also reduce testing delay (ie, the time duration between symptom onset and subsequent testing) by immediately referring people to testing facilities [5] and reduce the time of uncertainty by providing test results more quickly. Moreover, digital contact tracing might be more reliable than manual tracing, as it is not affected by recall bias [2], especially of casual contacts, and allows for anonymous contacts to be traced [5]. Finally, once released, contact tracing apps may reach the majority of the population within a short period of time. It is estimated that 3.6 billion people worldwide already had access to a mobile device in 2020 [6]. In Europe alone, 540 million people (72% of the population) are currently in possession of a functional smartphone [6].

### App Adoption and Effectiveness

Acceptance by the potential users is the major challenge for COVID-19 contact tracing apps reaching their potential. Simulation studies concordantly emphasize the importance of high rates of app adoption in the population. In a best-case scenario, Xia and Lee [2] found that 90%-95% of the population must use a contact tracing app to stop the spread of COVID-19 and allow normalcy without physical distancing. Based on data for the United Kingdom, Hinch and colleagues [4] found that the pandemic could effectively be suppressed if 80% of smartphone users, or 56% of the population, use a contact tracing app. Kretzschmar and colleagues [5] used data from the Netherlands and investigated different scenarios including adoption rates. In a best-case scenario in which the app adoption rate is 80%, almost 80% of forward transmission could be prevented. Interestingly, even with an adoption rate of 20%, contact tracing apps were more effective than manual tracing, and just 40% of the population needed to use the app to control the pandemic. Yasaka and colleagues [7] found the best results for an adoption rate of 75%, but even a rate 25% could provide some suppression of the infection curve. Similarly, Moreno López and colleagues [8] reported that a 30% adoption rate of tracing apps could be sufficient to reduce the pandemic to a manageable level if the dynamics of infection are moderate. Braithwaite and colleagues [9] presumed a quadratic relationship between a population’s app adoption rate (ie, 80% adoption rate) and associated reductions in transmission (ie, 64% of contacts notified). However, assuming an adoption rate of 53%, Kucharski and colleagues [10] suggested that manual tracing is more effective than digital tracing. It should be noted that no empirical data have yet been published, with all the aforementioned numbers merely the result of modeling studies whose parameter settings might be debated (eg, assumptions made by Kucharski and colleagues [10] about the capacity of manual tracing in high incidence situations might be too optimistic). In a recent combined simulation and observational study, Wymant and colleagues [11] found that every 1% increase in app adoption lead to a decrease of 0.8% up to 2.3% in infections. To summarize, these studies suggest that high adoption rates are vital for contact tracing apps to play a key role in fighting COVID-19 but that “lower numbers of app users [would] also have a positive effect” [4].

Initial studies on the intention to download and use a contact tracing app were encouraging. In a large multinational study in Western countries (France, Germany, Italy, the United Kingdom, and the United States), the intention to use a contact tracing app ranged from 68% to 75% [12]. In a Belgian study, 49% of participants intended to use a contact tracing app [13]. In a large study in Ireland, 83% claimed that they would either definitely or probably install a contact tracing app on their smartphone [14]. Additionally, in a Dutch sample, app adoption rates varied between 59% and 66%, depending on the app’s features [15].

Numerous factors have been investigated as potential predictors of app adoption. They have included potential users’ age, gender, comorbid illness, smartphone use, trust in the government, opt-in installation [12], belonging to a higher risk group, general trust in others, privacy concerns [16], the presence of serious health conditions, level of education, fear of infection, expected general adoption rate in the society [15], attitudes toward protecting family members and friends, feeling responsible to the community, COVID-19–related worry [14], perceived benefits, cues to use the app in the media, and self-efficacy toward properly using the app [13].

### Quality Characteristics

Although such personal characteristics, individual expectations, and societal variables are important, quality-related characteristics of contact tracing apps might also play an important role in their acceptance and adoption rates. According to Stoyanov and colleagues [17], a health app’s quality characteristics can be subcategorized into those pertaining to engagement, functionality, aesthetics, and information quality. These four dimensions of COVID-19–related app quality were investigated in an early review published by Davalbhakta and colleagues [18], focusing on India, the United States, and the United Kingdom. Most apps provided information on COVID-19 or included symptom checkers. In general, the quality of COVID-19 apps was above average, and apps were designed to achieve higher scores in functionality, while features to make apps engaging and important to the user were not as highly considered. Interestingly, adoption rate (measured through the number of app downloads as a proxy for adoption) was not correlated with app quality. For Europe, Davalbhakta and colleagues [18] claimed that apps generally focus on providing high quality information from credible sources but often lack creative and interactive methods to provide this information. At the time of their review, only two COVID-19 contact tracing apps from national health authorities had been launched in the European Union. Since then, almost all European countries have developed and launched COVID-19 contact tracing apps. To the best of our knowledge, their quality characteristics and actual adoption rates have not been yet systematically investigated.

The primary aim of this study is, therefore, to investigate the quality characteristics of European national COVID-19 contact tracing apps, in terms of their engagement, functionality, aesthetics, and information quality. A secondary aim is to examine quality characteristics and their associations with app adoption rates. Likewise, we will investigate adoption rates and associated numbers of confirmed COVID-19 cases as a measure of pandemic control. Furthermore, we also will analyze features embedded within COVID-19 contact tracing apps. Finally, based on our results and the identification of apps with the highest quality scores, we will provide recommendations for optimizing contact tracing apps and increasing adoption rates.

## Methods

### Search Strategy

Eligible countries were member states of the European Union, former members (the United Kingdom), and countries of the European Free Trade Association (Switzerland, Iceland, Norway, and Liechtenstein). These countries share similar standards of voluntariness of app use, data protection, and privacy regulations, namely the European General Data Protection Regulation (GDPR) or regulations that are equivalent to these standards. Further inclusion criteria were a tracing app launched by a national health authority, voluntary app use, unrestricted access to the app, English version available, and app launch at least 4 weeks before the review was conducted. To identify all relevant national COVID-19 contact tracing apps, an English Google search was performed using the keywords “corona tracing app,” “corona app,” and “covid app” combined with the name of each country. For the United Kingdom, we considered England, Scotland, Wales, and Northern Ireland separately. For some countries, no national tracing apps were identified directly; therefore, the search was repeated in each country’s main language using translation software. The apps were then downloaded through the Google Play Store or Apple App Store.

### Interdisciplinary Quality Rating

Quality characteristics were assessed with the Mobile App Rating Scale (MARS) [17] using the German version [19]. The MARS quality section consists of four subscales: engagement, functionality, aesthetics, and information quality. These scales collectively contain 19 questions that reviewers are asked to respond to using a 5-point response scale (1: inadequate; 2: poor; 3: acceptable; 4: good; 5: excellent). One item directed at the level of evidence of treatments (item 19) was excluded. For each scale, the mean score was calculated, ranging from 1 to 5. The MARS has been shown to exhibit very good internal consistency (α=.90) and interrater reliability (intraclass correlation coefficient [ICC] 0.79) [17].

Four reviewers were used in this study: two researchers with a background in e–mental health and two researchers from the human–computer interaction field. All experts rated the apps independently of each other, after each rater finished the rater training proposed by Messner and colleagues [19] and practiced ratings on 3 apps for depression. The scores were combined and ICCs calculated for each app. In cases of ICCs below 0.75, discrepancies in ratings were discussed until consensus about the rating was reached, as proposed by Messner and colleagues [19]. For the ratings, two devices operating with iOS and two with Android were used.

### Adoption Rates

All app developers were asked, via email, to provide the numbers of downloads. In cases of no reply, a reminder was sent after 1 week. If answers from developers could not be attained personally, the number of downloads was extracted from published statistics by national health authorities, app developers, or other publicly available sources. We calculated the percentual of the download count within the population in each country to obtain the overall adoption rate. The total download numbers, numbers of inhabitants, and data of retrieval are available in Multimedia Appendix 1.

### Infection Rates

For each country, the number of infected people were obtained from publicly available data from the COVID-19 Dashboard by the Center for Systems and Engineering at Johns Hopkins University [20]. The time span from app release to the date of measuring adoption rate was considered. Numbers are reported in Multimedia Appendix 1.

### Statistical Analysis

A mixed methods approach was used for this review. For the MARS total score and each subscale, the mean score was calculated and reported, ranging from 1 to 5. ICCs were calculated using a two-way mixed effects average measures model with absolute agreement [17]. To explore associations between contact tracing app quality characteristics and adoption rates, scatterplots and trend lines were presented together with univariate ordinary least squares (OLS) and Bayesian regression analysis [21]. Due to the expected low sample size of European contact tracing apps, any significance testing and relying on *P* values are highly likely to miss existing associations (type II error). Therefore, we focused on effect sizes and reported all associations exceeding a medium effect size, as recommended by Cohen [22].

Additionally, we calculated adoption rates for best-case scenarios (best-case adoption rate [BCAR]), assuming that app quality characteristics were optimized (five points on the MARS). The same analyses were conducted to investigate associations between adoption rates and measures of pandemic control.

As part of our qualitative analysis, all raters were instructed to identify features of the tracing apps that, in their opinion, might increase user engagement and adoption rates. Features were identified by all raters and then discussed and classified by all authors. As a result, all apps were analyzed with respect to the features they offered. Additionally, apps with high scores on the various MARS items were presented as examples of best practice. Finally, MARS ratings, identified features, and best practice guided recommendations for optimizing acceptance and adoption of COVID-19 contact tracing apps are proposed.

## Results

### Search

**Figure 1:**
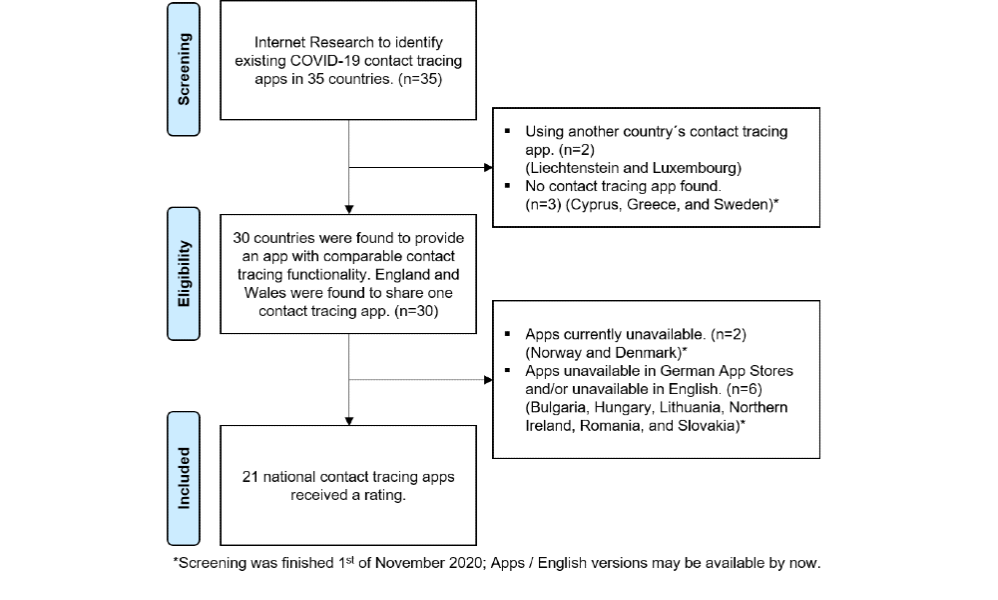
App selection process flowchart.

We identified 21 contact tracing apps that we then included in our quality review. For a detailed description of the search process, see Figure 1.

### Interdisciplinary Quality Rating

For 17 apps, ICCs between 0.75 and 0.89 were detected, indicating good interrater reliability, while excellent interrater reliability (ICC>0.90) was observed for 2 apps. Due to low ICCs, ratings for 2 apps needed to be discussed, resulting in moderate ICCs of 0.70 (France) and 0.65 (Poland) [23].

**Figure 2:**
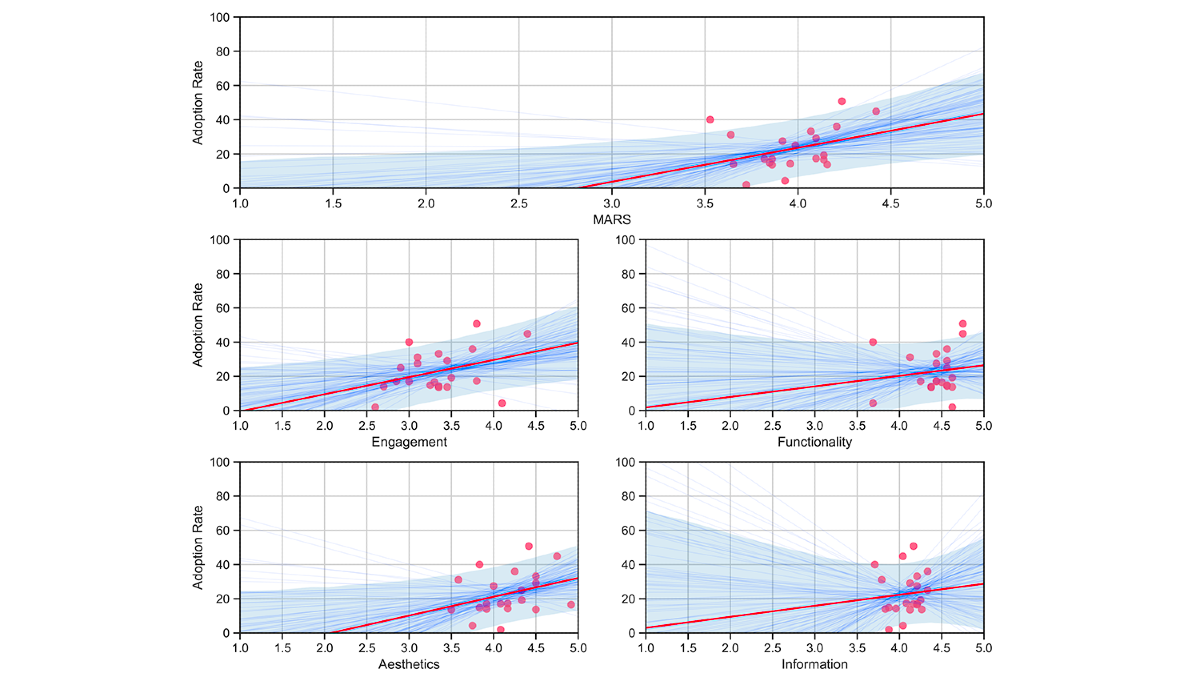
Bayesian regression with trend lines and 80% CIs (blue shaded area) for app characteristics and adoption of COVID-19 contact tracing apps. MARS: Mobile App Rating Scale.

In general, the average MARS total score was 3.97, indicating above average app quality, with mean scores ranging from 3.53 to 4.42 (see Table 1 and Figure 2). The Irish app was rated best (MARS ∅=4.42), almost 1 SD above the app with the second highest rating (Finland; MARS ∅=4.24). With regard to app quality characteristics, functionality, aesthetics, and information quality scored over 4 points. High scores for information quality and functionality were especially associated with low variance, indicating small differences between the apps (Table 1). The scores for engagement were lower, on average, and the differences between apps larger. Although high scores for functionality reflect a ceiling effect and scores for aesthetics and information quality a close-to-ceiling effect, lower scores for engagement indicate opportunities for improvement.

**Table 1:** MARS rating scores on all apps (N=21).

Country	App name	MARS^a^ total	Engagement	Functionality	Aesthetics	Information	Downloads, n	Adoption rate (%)
All, mean (SD)	N/A^b^	3.97 (0.22)	3.34 (0.45)	4.43 (0.28)	4.16 (0.37)	4.09 (0.18)	97,097,051	22.92 (12.51)
Austria	Stopp Corona App	3.85	3.25	4.56	3.83	3.88	1,312,063	14.81
Belgium	Coronalert	4.14	3.50	4.63	4.33	4.25	2,200,000	19.20
Croatia	Stop COVID-19	3.72	2.60	4.63	4.08	3.88	78,534	1.93
Czech Republic	eRouška	3.86	3.35	4.38	3.50	4.13	1,443,691	13.56
England/Wales	NHS COVID-19	4.21	3.75	4.56	4.25	4.33	20,361,253	35.97
Estonia	HOIA	3.86	3.00	4.44	3.92	4.17	224,833	17.31
Finland	Koronavilkku	4.24	3.80	4.75	4.42	4.17	2,800,000	50.74
France	Tous Anti-COVID	4.10	3.80	4.44	4.17	4.08	11,600,000	17.31
Germany	Corona-Warn- App	4.10	3.45	4.56	4.50	4.13	24,200,000	29.15
Iceland	Rakning C-19	3.53	3.00	3.69	3.83	3.71	142,796	40.00
Ireland	COVID Tracker Ireland	4.42	4.40	4.75	4.75	4.04	2,200,000	44.86
Italy	Immuni	4.14	3.30	4.50	4.92	4.21	10,000,000	16.57
Latvia	Apturi COVID	4.16	3.45	4.63	4.50	4.26	263,848	13.74
Malta	CovidAlert Malta	3.82	2.85	4.25	4.08	4.21	84,210	17.06
Netherlands	Corona Melder NL	3.99	2.90	4.56	4.33	4.33	4,321,443	25.01
Poland	STOP COVID ProteGO Safe	3.93	4.10	3.69	3.75	4.04	1,637,927	4.31
Portugal	STAYAWAY COVID	3.92	3.10	4.44	4.00	4.21	2,822,522	27.47
Scotland	Protect Scotland	3.64	3.10	4.13	3.58	3.79	1,700,000	31.12
Slovenia	#OstaniZdrav	3.96	3.35	4.56	4.17	3.96	297,000	14.27
Spain	Radar COVID	3.65	2.70	4.38	3.92	3.83	6,571,600	14.00
Switzerland	SwissCovid	4.07	3.35	4.44	4.50	4.21	2,835,331	33.18

^a^MARS: Mobile App Rating Scale.

^b^N/A: not applicable.

### Adoption Rate

European COVID-19 contact tracing apps had an average population adoption rate of 23%, with high variation between countries, indicated by an SD of 12.5%. Taking different population sizes into account, the overall adoption rate was estimated at 22%. Adoption varied between the highest rates for Finland (51%), Ireland (45%), and Iceland (40%), and the lowest for the Czech Republic (14%). Poland (4%) and Croatia (2%) were considered low adoption outliers. Data on app release are summarized in Multimedia Appendix 1.

### App Quality Characteristics and Adoption Rates

**Figure 3:**
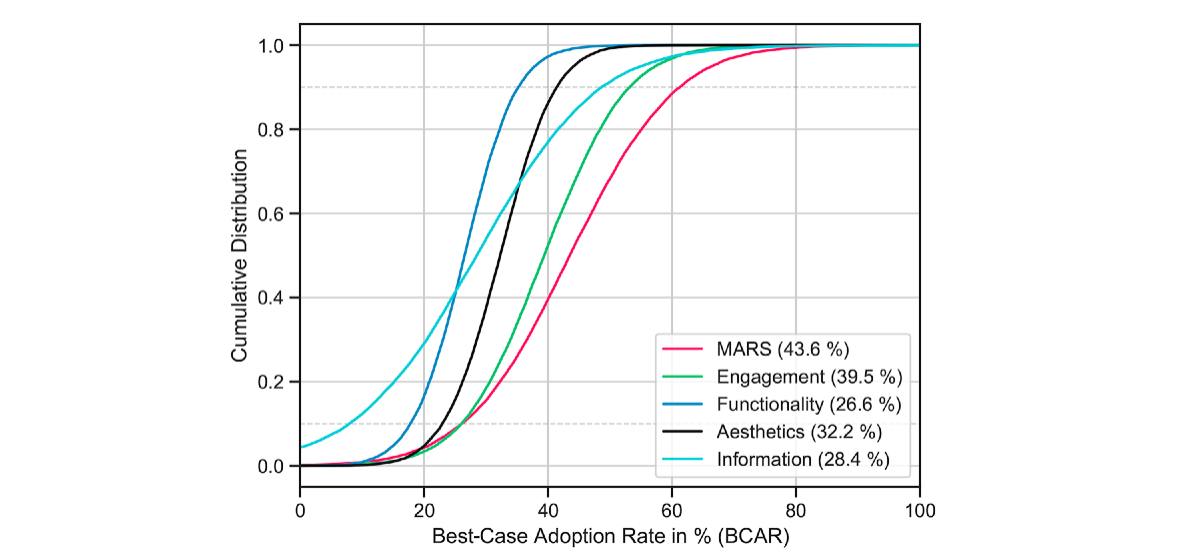
Cumulative distribution of the BCAR; numbers in brackets indicate the mean BCAR for specific app characteristics. BCAR: best-case adoption rate; MARS: Mobile App Rating Scale.

The MARS total score and two dimensions of quality characteristics exceeded the minimum threshold for an intermediate strength correlation: general app quality (MARS total score; R=0.35), engagement (R=0.35), and aesthetics (R=0.31). To calculate the BCAR, we conducted a univariate Bayesian linear regression using 20,000 Markov chain Monte Carlo draws for each app characteristic. Results are presented in Table 2 and Figure 2. This analysis yielded one BCAR distribution per app characteristic (Figure 3), resulting in a mean BCAR of 44% for apps with maximum general app quality, 40% for apps designed in the most engaging way, and 32% for apps with maximum scores for aesthetic design. Among app quality characteristics with high average scores—functionality and information quality—no associations exceeded the minimum threshold.

**Table 2:** Regression analysis app characteristics correlated with moderate-effect sizes for adoption of COVID-19 tracing apps (results for Bayesian regression are based on 20,000 draws).

Predictor			R		Intercept, mean (SD)		*t* test (*df*)		Slope, mean (SD)		*t* test (*df*)		BCAR^a,b^ 10% percentile		BCAR		BCAR 90% percentile
MARS^c^ total score			0.349		–55.68 (50.43)		1.10 (19)		19.82 (12.69)		1.56 (19)		25.28		43.56		61.21
**Quality characteristics (dimensions)**																	
	Engagement	0.354		–10.53 (21.07)		0.50 (19)		10.02 (6.25)		1.60 (19)		25.77		39.54		53.69	
	Functionality	0.139		–4.58 (45.83)		0.10 (19)		6.21 (10.34)		0.60 (19)		17.71		26.56		34.78	
	Aesthetics	0.314		–22.36 (33.61)		0.67 (19)		10.90 (8.04)		1.36 (19)		22.96		32.17		41.18	
	Information	0.084		0.33 (68.30)		0.00 (19)		5.53 (16.71)		0.33 (19)		8.14		28.37		48.16	
**Specific quality characteristics (items)**																	
	Interest (item 2)	0.405		3.48 (11.07)		0.31 (19)		5.98 (3.29)		1.82 (19)		25.37		33.32		41.17	
	Interactivity (item 4)	0.300		–7.31 (23.87)		0.31 (19)		9.51 (7.45)		1.28 (19)		22.87		40.18		57.43	
	Target group (item 5)	0.488		–37.72 (26.19)		1.44 (19)		13.99 (6.02)		2.32 (19)		26.12		32.21		38.20	
	Graphics (item 11)	0.324		–36.04 (40.44)		0.89 (19)		13.00 (8.89)		1.46 (19)		22.41		28.86		35.22	
	Accuracy of app description (item 13)	0.424		–105.55 (66.47)		1.59 (19)		26.77 (13.84)		1.93 (19)		23.29		28.30		33.27	
	Quantity of information (item 16)	0.413		–10.72 (38.72)		0.28 (19)		7.83 (8.99)		0.87 (19)		19.41		28.30		37.04	

^a^BCAR: best-case adoption rate.

^b^BCARs are assuming maximum MARS scores.

^c^MARS: Mobile App Rating Scale.

For 18 specific quality characteristics, six MARS items were correlated with higher adoption rates with at least a medium level effect (Table 2). Higher ratings for interesting use facilitating frequent engagement correlated with higher adoption (item 2; R=0.41) and an above average BCAR of 33%. More interactive apps—allowing user input, providing feedback, and containing prompts (item 4)—were directly correlated with app adoption (R=0.30; BCAR 40%). Likewise, appropriate app design for the target group (item 5) was correlated with the app adoption rate (item 5; R=0.49, BCAR 32%). These three specific app characteristics are part of the engagement dimension. Item 11 considers the quality of graphics and visual design of buttons, icons, menus, and content as part of the aesthetic dimension. Higher scores were correlated with higher adoption rates (R=0.32; BCAR 29%). The accuracy of the app description in app stores (item 13) and the quantity of information (item 16) represent facets of the information quality dimension. A highly accurate description of the components and functions of the app was correlated with higher adoption rates (R=0.42; BCAR 28%). Likewise, comprehensive and concise information with links to more information and resources was correlated positively with adoption (R=0.41; BCAR 28%). The regression model for the target group was the only one with significant effects.

### App Adoption and Infection Rates

**Figure 4:**
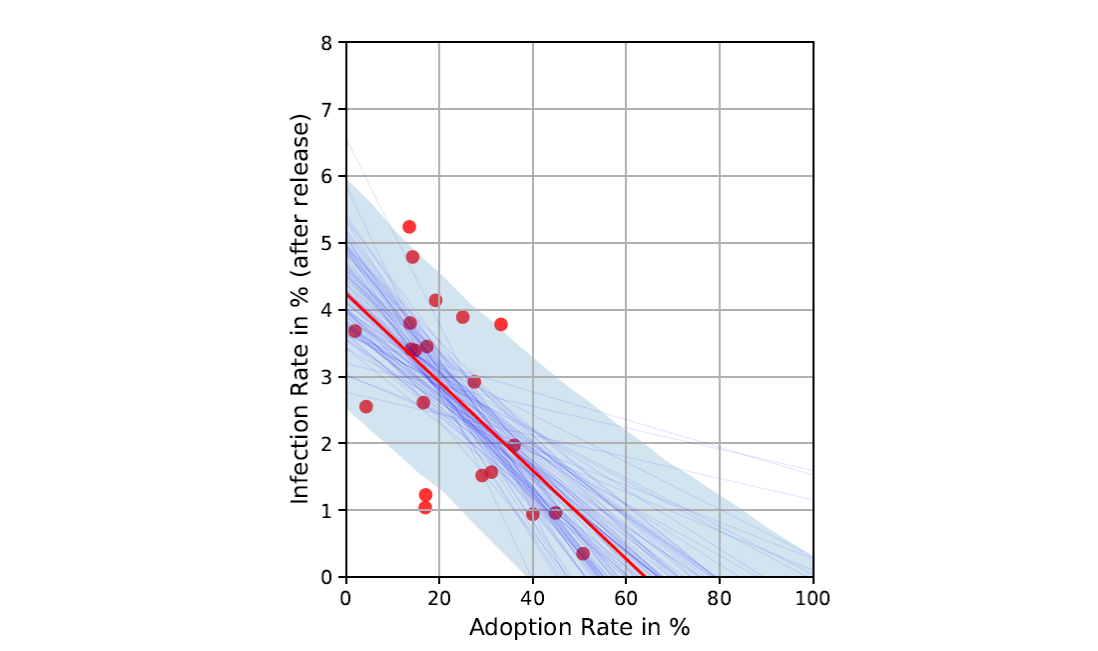
Bayesian regression with trend lines and 80% CI for app adoption of COVID-19 contact tracing apps and infection rate after app release.

Next, the associations between app adoption rates and infection rates due to COVID-19 were investigated (see Figure 4). Results for Bayesian regression based on 20,000 draws suggested an inverse correlation between app adoption and infection rates after app release: the higher the adoption rate, the lower the rate of infections (r=–0.606; *P=*.004). Based on numbers at the time of assessing adoption (mean 22.92) and infection (mean 3.05) rates, the results indicated that a 1-point increase in adoption is associated with a 0.07 percentage points decrease in infection rate, representing a change in infection rate of 2.30%. According to the OLS regression (y = 4.23 + –0.07 * x), a zero rate of infections (trend line intersects x-axis) is reached if the adoption rate equals 60.43%. The SD of the intercept was 0.57 (*t*_19_=7.36), and for the slope, it was 0.02 (*t*_19_=2.99). Excluding low adopting outliers, the effects were even more pronounced for infection rates (r=–0.656; *P=*.002).

### Features of COVID-19 Contact Tracing Apps

On qualitative analysis, various features of the COVID-19 contact tracing apps were identified that might contribute to higher adoption rates. This section gives an overview of features implemented by presenting best practice examples. We focus on features that contributed to higher ratings in each MARS dimension.

#### Engagement

Most features aimed to increase users’ engagement with the app. Table 3 provides an overview of features believed to enhance engagement beyond the core functionality of contact tracing. Apps with high scores on engagement—like those in Ireland, Poland, Finland, France, and England and Wales (see Table 1)—provided best practice examples.

**Table 3:** Features of COVID-19 contact tracing apps contributing to engagement.

Categories and features^a^			Countries																																								
			AT^b^		BE^c^		CH^d^		CZ^e^		DE^f^		EE^g^		EN^h^		ES^i^		FI^j^		FR^k^		HR^l^		IS^m^		IE^n^		IT^o^		LV^p^		MT^q^		NL^r^		PL^s^		PT^t^		SC^u^		SI^v^
**Symptoms**																																											
	Symptom checker^w^	✓^x^		—^y^		—		—		—		—		✓		—		✓^z^		—		—		—		—		—		—		—		—		✓		—		✓^z^		—	
	Symptom diary^aa^	—		—		—		—		—		—		—		—		—		—		—		—		✓		—		—		—		—		✓		—		—		—	
**Regularly updated information provided within the app**																																											
	Statistics on COVID-19^ab^	—		✓		✓		✓		—		—		—		—		✓^z^		✓		—		—		✓		—		✓		✓^z^		—		—		—		—		—	
	App use^ac^	—		—		✓		✓		—		—		—		—		—		✓		—		—		✓		—		—		—		—		—		—		✓		—	
	Public health instructions/restrictions^ad^	—		—		—		—		—		—		—		—		✓^z^		✓		—		✓^z^		—		—		—		✓^z^		—		✓^z^		—		—		—	
	Burden on health care system^ae^	—		✓		—		✓		—		—		—		—		✓^z^		✓		—		—		✓		—		—		—		—		—		—		—		—	
**Individualization**																																											
	Assigning personal data^af^	—		—		—		—		—		—		✓		—		—		—		—		✓		✓		✓		✓		—		—		✓		—		—		—	
	Updates on assigned region^ag^	—		—		—		—		—		—		✓		—		—		—		—		—		—		—		—		—		—		—		—		—		—	
**Tracing**																																											
	Control over tracing activity^ah^	✓		✓		✓		✓		✓		✓		✓		✓		✓		✓		✓		—		✓		✓		✓		✓		—		✓		✓		✓		✓	
	Contact diary^ai^	—		—		—		—		—		—		—		—		—		—		—		—		—		—		—		—		—		✓		—		—		—	
	Venue check-in^aj^	—		—		—		—		—		—		✓		—		—		—		—		—		—		—		—		—		—		—		—		—		—	
**Communication**																																											
	Chat^ak^	—		—		—		—		—		—		—		—		—		—		—		✓		—		—		—		—		—		—		—		—		—	
	COVID-19–related helplines^al^	—		—		✓		✓		—		—		—		—		—		—		—		✓		—		—		—		—		✓		✓		—		—		—	
	Sharing^am^	✓		✓		✓		✓		✓		—		—		—		—		✓		—		—		✓		✓		✓		—		✓		—		✓		✓		✓	

^a^The specified functions represent the status as of December 5, 2020. App development is dynamic and additional features may have been released in the meantime (eg, a contact diary and daily statistics have been added in the German app).

^b^AT: Austria.

^c^BE: Belgium.

^d^CH: Switzerland.

^e^CZ: Czech Republic.

^f^DE: Germany.

^g^EE: Estonia.

^h^EN: England/Wales.

^i^ES: Spain.

^j^FI: Finland.

^k^FR: France.

^l^HR: Croatia.

^m^IS: Iceland.

^n^IE: Ireland.

^o^IT: Italy.

^p^LV: Latvia.

^q^MT: Malta.

^r^NL: Netherlands.

^s^PL: Poland.

^t^PT: Portugal.

^u^SC: Scotland.

^v^SI: Slovenia.

^w^Symptom checker: symptom checker is used only if required.

^x^Included in this app.

^y^Not included in this app.

^z^Indicates that features are displayed on an external website.

^aa^Symptom diary: symptoms are monitored daily and symptom history is saved.

^ab^Statistics on COVID-19: numerical information regarding infections, deaths, tested, etc.

^ac^App use: numbers of app downloads, transferred positive COVID-19 tests, warnings from the app, etc.

^ad^Public health instructions/restrictions: app links to current information on the pandemic, for example, on how to protect oneself from the virus or which restrictions are currently in place.

^ae^Burden on health care system: numerical information on available intensive care units, numbers of COVID-19 cases hospitalized, etc.

^af^Assigning personal data: potential to provide personal data (telephone number, nickname, postal code).

^ag^Updates on assigned region: updates on the status of assigned region of residence.

^ah^Control over tracing activity: potential to disable tracing in app settings.

^ai^Contact diary: users can document their contacts.

^aj^Venue check-in: using a Quick Response code, users check into venues.

^ak^Chat: app provides a chat function.

^al^COVID-19–related helplines: app provides telephone numbers to seek help (medical).

^am^Sharing: app has a (prominently displayed) button so the app can be shared.

#### Interest

Several apps provided a *symptom checker.* They included a series of COVID-19–specific questions and provided individual feedback and recommendations. Typically, users could assess the occurrence and intensity of symptoms and receive recommendations for appropriate action (eg, scheduling an appointment at a test center). Users were free to check symptoms as often as they wished. Some apps addressed the benefits of checking symptoms by providing a link to an external website. Accordingly, users had to leave the app, and those apps could not make use of any data on the external website. However, most symptom checkers saved symptomatic data and included a history of symptoms within the app. In this case, a *symptom diary* was coded. The symptom diary enabled users to monitor symptoms that were displayed using a numeric and text-based design. Graphic displays to present the course of symptoms were not observed. Noteworthy is that the checking feature implemented in the Polish app was a combined *risk group checker* and symptom checker. Therefore, users must indicate age and any pre-existing illness (eg, obesity along with current symptoms), and feedback probably was provided by taking all this information into account. A separate risk group checker informing users about, for example, any increased risk of severe COVID-19 symptoms, was not identified in any app. Besides the symptom diary, a *contact diary* was observed in 1 app (Poland). Users can take written notes on each contact, which helps them to remember contacts and give more detailed information in case of infection. Other modes of contact diary input or output—like speech, picture, or location-based notes—were not found. All these checking and monitoring features lead to higher scores for engagement, as they provide added value and invite users to interact with the app more often.

Features providing new and relevant information contributed to higher scores in engagement as well. First, several apps informed the users about current numbers of newly infected people and the number of infected people who had died (see Table 3). Second, some apps provided information on public health measures, like current restrictions during a lockdown. Third, users receive information on the capacity of and burden on the health care system (eg, current number of hospitalized patients or occupied beds in intensive care units, as done with the French app). Fourth, some have features informing users about the number of downloads, thereby informing them about the size of the app-using community and motivating them to share the app with others. Fifth, a few apps additionally informed users about the protective behaviors of other users (eg, number of shared positive test results; see Swiss app) or about others’ self-protective behaviors, like the number of users that conducted a symptom check that day (see Irish app). Such features were rated positively, as they can strengthen a sense of belonging to a community of people who take the pandemic seriously and care for one another. However, it should be noted that apps provided this information differently: sometimes directly included in the app, while others provided a link to an external website, thereby, interrupting users’ engagement with the app.

Although paper-and-pencil lists were used in several countries to document visitors (eg, museums) or customers (eg, restaurants), the *venue check-in* (see English and Welsh app) offered a digital solution as part of their tracing app by scanning the QR (Quick Response) codes of pertinent venues. As all visitors are registered with a time stamp, it is possible to exchange information on infections quickly and reliably.

#### Customization

Several features were identified that contributed to higher customization ratings. First, with regard to visual customization, the Portuguese app offered users the opportunity to switch between different themes: dark mode, light mode, and automatic. In this way, users can choose the mode that appeals most to them, as opposed to most other apps, which were designed using a one-size-fits-all strategy. Second, there was one example of a customized welcome message (Polish app); with this, users could voluntarily assign themselves a nickname to receive a personalized welcome while using the app. Third, some apps provide regional customization, like the English and Welsh app, so users can receive news about the status of COVID-19 in their own region of residence. To facilitate the identification of COVID-19 hot spots, the Italian app asked users to indicate their region of residence. Fourth, users can customize the channel of support by voluntarily providing their phone number as a callback option (Irish and Latvian apps). Fifth, most apps offer to customize tracing functionality, meaning that users can pause contact tracing. Most of these customization options were voluntary. However, to be able to use the app, it was sometimes mandatory for users to indicate their postal code (England and Wales) or provide their telephone number (Iceland).

#### Interactivity

High interactivity facilitates interactions between users or considers the interaction between the user and the app. First, interactive symptom diaries and checkers were rated higher than noninteractive versions of these features (eg, the app links to a symptom checker on an external website). A high level of interactivity also is achieved if symptoms can be checked within the app and feedback provided based on the results, for example, with recommendations regarding self-isolation and taking a test (English and Welsh app) or friendly, encouraging feedback when no symptoms are reported (Irish app). Likewise, the interactive risk group checker provided individual feedback. Lower interactivity was deemed present in apps that merely included links to external websites to assess potential symptoms of COVID-19. Second, features that allow users to share the app with their family and friends in an easy and comfortable way contributed to higher ratings for interactivity (eg, the Dutch app). Third, 1 app provided users the option of interacting directly with health officials via chat (Iceland).

#### Target Group

Adequate consideration of the target group relates to intended users’ sociodemographic characteristics, preferences, and needs. First, all reviewed apps were rated to be designed for the average user and the whole population, irrespective of user age, gender, or socioeconomic background. Apps received higher scores when the language used was easy to understand. Within most apps’ illustrations, the icons and images supported textual information and contributed to better understanding. Likewise, almost all the apps were designed according to current trends and standards. Noteworthy is that illustrations within the English and Welsh app were designed to represent a diverse society with people from different cultures. Second, the target group can also be characterized by their needs and expectations (eg, the need for predictability, users feeling well prepared when they receive an alert, or using the app to share one’s own positive test result with others). The Dutch app provides a good example for a preview on how the app will alert the user in the event of a detected encounter with a person who has tested positive. To do so, this app uses explanatory text paired with graphic support.

#### Functionality, Aesthetics, and Information

In general, the functionality, aesthetics, and information quality of all the apps were rated highly, with minimal variance between the ratings. Therefore, only a few features were identified that could make a difference and potentially increase users’ adoption of the app. According to their MARS ratings, the rater team identified best practice examples of app features for each dimension: the Finish and Irish apps provide good examples for functionality, the Italian for aesthetics, and the Dutch and English/Welsh apps for information.

High scores for functionality were achieved by most of the apps, as they performed very well. Their structure was easy to understand because content areas were often clearly separated by card-based layouts and marked with headings. Additionally, all the apps were easy to use. In terms of navigation, some differences appeared. For example, tab navigation with icons and text in the footer and metanavigation integrated into the header was rated highly, as this configuration allows easy orientation (ie, Irish and Portuguese apps).

Most apps were rated highly with regard to aesthetics, with the graphic design of most corresponding to design trends of the years 2020 and 2021 (based on research by creative platforms, creative experts, and agencies), thereby making them attractive for most people. All apps used sans serif fonts and almost all preferred illustrations over photos. Most apps and almost all illustrations appeared in flat design, sometimes in combination with light background gradients or background circles. The character designs often appeared on monochrome backgrounds (eg, Belgium, Germany, and Slovenia) in analogue color schemes (eg, Italy and Finland) or with complementary color schemes (eg, Germany and the Netherlands), presumably to focus on the content or the message of the illustrations, rather than to illustrate the app’s functionality. Disproportionate body parts embodied the character design of some apps (eg, Italy), for example, with small heads on large bodies with large limbs, while detailed facial features were largely avoided. The modern line art style was used especially for icons in most apps (eg, Finland’s navigation tabs). Meanwhile, with the Estonian app, this style was used for the entire app’s appearance. The use of curved lines was an exception only observed with the Latvian app. Buttons often appeared haptic, with drop shadows compared to illustrations, making it easier for users to identify elements of the interface that they can interact with.

Apps with the highest aesthetics ratings used different font styles with high contrast and strong, saturated colors for components of the interface design. Visual information groups were formed using the law of proximity, in the form of cards, spaces between components, and white space in general. Based on our assessment of the apps, visual appeal was mostly characterized by flawless graphics and a uniform and professional design. Small animations bring graphics to life (eg, Italy), stimulating positive feelings in users.

High ratings for information quality were achieved by high-level accuracy in app descriptions in app stores, reflecting the app’s actual functionality. Likewise, the apps’ goals were well described in the stores or within the apps. Some differences were observed in the extent to which illustrations and visualizations (tables, images, graphics) were used to deliver information. Generally, apps with more illustrations and visualizations were rated higher. Credibility of information was rated high, as all apps were published by the health authorities in their country of use. Noteworthy is the Dutch app, a good example of providing concise and helpful information on how to use the app. For example, users can easily understand how the app will warn them if they ever come in contact with a person testing positive for COVID-19.

## Discussion

### General Findings

This study investigated quality characteristics among European COVID-19 contact tracing apps and explored these characteristics’ associations with user adoption rates. In general, scores for functionality, aesthetics, and information quality were high, and differences between the apps were minor. However, the quality of features aiming to increase users’ engagement with the app was lower, on average, with substantial differences between the apps that reflected large differences in adoption rates. Alongside quality ratings, we identified engagement-friendly features implemented in tracing apps that contribute to the higher quality of those apps, may guide further development, and could increase the acceptance and adoption of contact tracing apps in the general population. These engagement-friendly features are presented within nine recommendations for an engagement-oriented design for COVID-19 contact tracing apps.

### Findings in Context

Comparing the level of quality observed in our study to the earlier review by Davalbhakta and colleagues [18], there is increased quality of the COVID-19 contact tracing apps, with an overall MARS rating 0.52 points higher, a 13% increase. Especially with regard to information quality (+0.83) and aesthetics (+0.68), apps achieved higher ratings, while scores for functionality were almost unchanged. This overall improvement is probably because the apps assessed in this study were in place later in 2020, meaning that developers simply had more time to improve features like aesthetics and engagement beyond tracing core functionality. Differences in information quality might be explained by all the apps in this study having been released by the countries’ own national health authorities. Interestingly, the relative order in tracing apps’ quality—with functionality scoring highest, followed by aesthetics and information quality, and engagement lowest—found both in our study and the study conducted by Davalbhakta and colleagues [18] has also been observed in apps for other health conditions, like rheumatism [24], heart failure [25], and posttraumatic stress disorder [26], and for health behaviors, like physical activity [27], and might indicate a general underemphasize of engagement-friendly designs for health-targeted apps. Overall, adoption rates were substantially lower than anticipated, based on the results of early acceptability studies [12,15], and lower than several simulation studies found to achieve pandemic control [2,4,7]. However, the observed average adoption rate was close to 25%, which was a lower threshold for a suppressive effect of tracing apps on the infection curve in the study of Yasaka and colleagues [7], while Wymant and colleagues [11] highlighted that every 1% increase in app adoption leads to a meaningful decrease in infections. With regard to acceptability studies, the discrepancy might be explained by the gap between intention to use a tracing app and actual behavior, a phenomenon known as the intention–behavior gap [28]. Another explanation is the reliance on nonrepresentative samples and the use of digital surveys that might be preferred by people with positive attitudes toward digital apps [29].

### App Design and App Adoption

Low adoption rates may also be the result of app design. To the best of our knowledge, this is the first study to investigate the association between app quality characteristics and actual adoption rates for national COVID-19 contact tracing apps. Bayesian regression analyses suggest that adoption rates can be increased by optimizing app quality characteristics. In our analysis, optimizing engagement features appeared to be associated with higher adoption rates, relative to strategies focused on functionality, aesthetics, or information quality. This finding has several implications. First, one promising strategy, from an app design perspective, appears to be to focus on engagement. Second, the app’s level of engagement is a modifiable factor that can quickly be made the target of developers and public health actions. Other factors that are associated with the acceptance of tracing apps are either not or minimally modifiable, like potential users’ age, gender, comorbidities, medical preconditions, level of education, household income, and trust in the government [12,30]. Third, it is not enough for tracing apps to trace and warn users properly. They should also be designed in a way that makes them interesting to use, stimulate repeated engagement with the app, offer customization and tailoring to personal preferences, allow user input, and provide feedback. Fourth, the predicted adoption rate in the case of optimal engagement was 40%. This potential increase is comparable or even larger than the 17% increase in adoption recently identified for monetary incentives for installing a COVID-19 contact tracing app [30]. Although it might be speculated that such different adoption-increasing interventions have an additional effect on adoption rates, a compensatory effect is possible. Finally, given the importance and potential impact on user rates of engagement-friendly app designs, this topic might deserve as much attention as data security and privacy issues.

### App Adoption and Infection Rates

Another important finding of this study was the association between higher app adoption rates and lower infection rates. As association is not necessarily causation and confounders have to be considered, results should be discussed and interpreted from different perspectives. First, following the line of reasoning that using tracing apps leads to lower infection, it is of interest that the predicted adoption rate of 60% in this empirical observational study required achieving a zero infection rate that was between the adoption rates reported for simulation studies by Hinch and colleagues [4] and Xia and Lee [2]. Congruent with simulation studies, our results may indicate a beneficial effect of COVID-19 contact tracing apps, in terms of controlling the pandemic. Similarly, in a combined empirical observational and modeling study, Wymant and colleagues [11] found that app adoption as measured by downloads is an indicator of active use, and higher app use leads to lower infections. Interestingly, compared to the findings of Wymant and colleagues [11], in our study, we observed almost the same effect of app adoption, namely, a 1% increase in app adoption was associated with a 2.3% reduction in infections. Second, the association might also be explained by reverse causality. Growing infection rates (eg, in the beginning of the second wave) could trigger fear, which in turn may increase app adoption. Building on the well-investigated effects of fear on health behavior change, it is possible that some individuals adopted tracings apps as a consequence of an increased perceived threat. However, it was found that the effect of fear on behavior is rather limited [31], and sometimes fear could also lead to undesired consequences (eg, avoidance) [32]. Therefore, the association between adoption and infection may partly but not largely be explained by reverse causality. In line with our results on the importance of engagement-friendly app design, it is also possible that spikes in incidences may raise awareness for the existence of tracing apps and motivate individuals to download apps given the precondition that apps are perceived as beneficial and engaging. Third, it seems likely that third variables play a considerable role in explaining the overserved association, as we found no country using tracing apps as the only nonpharmaceutical measure. In fact, they are used in concert with a multitude of other nonpharmaceutical interventions [33]. For example, app users are also more likely to adhere to other public health measures, like adherence to social distancing recommendations, wearing a mask, and washing hands [30]. Accordingly, tracing app use probably represents one specific health behavior that is part of a broader set of protective attitudes and behaviors for self and others. Depending on the respective national policy, app use could facilitate and boost other measures (eg, testing as a consequence on exposure notification or results from an app symptom checker) [11]. In this case COVID-19 tracing apps could also be considered as a hub for a multitude of measures to fight the pandemic. Accordingly, it is difficult to disentangle the unique contribution of tracing apps, and interactions between measures should be considered when interpreting the considerable strong association between app adoption and infection.

### Features and Recommendations

The features identified in this study using MARS as a guiding framework include those mentioned in prior reviews, such as self-monitoring, symptom checking [18,34], and contacts to helplines [35]. Although information or news related to COVID-19 represent broad categories [18,35], this study provides a more detailed description of existing features; for example, by differentiating between information content and purpose. Moreover, new features were observed like venue check-in, contact diary, and providing users control over tracing activity within the app. Generally, we observed a tendency toward multifeature COVID-19 contact tracing apps. Although apps evaluated earlier generally were limited to specific functionalities and features (eg, separate apps for tracing, symptom checking, and information dissemination [18]), apps reviewed later in 2020 seem to incorporate different features, thereby making the COVID-19 contact tracing app a broader hub for preventing COVID-19 transmission. Likewise, Collado-Borrell and colleagues [35] found that most apps had more than one purpose.

Based on best practice examples and the features observed in existing apps, recommendations were derived to foster an engagement-oriented app design (see Textbox 1).

**Recommendations for an engagement-oriented design for COVID-19 contact tracing apps.**

- Strive for a multifeature tracing app and avoid a trace-and-warn functionality only app. Offer additional features that make daily living during the pandemic more convenient and secure (eg, by replacing paper contact lists with a digital venue check-in).
- Make the app interesting and relevant to stimulate daily use by providing useful information (eg, current infection rates; intensive care unit capacities; lockdown rules; and legal regulations pertaining to schools, work, commuting, etc)
- Make the app as regionally targeted as possible, indicating the region most relevant to each particular user’s daily life, since public health measures (eg, regional lockdown rules) might only apply to certain regions. Matching the app’s level of regionality to local applied public health measures might increase user acceptance and adherence.
- Address the need of relatedness and create a virtual community of people who are committed to protecting themselves and others (eg, by making sharing the app as easy and obvious as possible). Provide feedback on other users’ protective behaviors toward self and others (eg, indicating the number of people using the app or sharing a positive test result).
- Include interactive and personalized features, like risk group and symptom checkers, and a symptom diary with individual feedback and recommendations. Such features encourage protective behaviors for both self and others, including early testing. They provide additional beneficial features to the user, stimulating frequent use, while personalization makes the app more relevant to users.
- Account for suboptimal connectivity between app users and extend the benefits of the digital solution to those not using a tracing app. For example, a venue check-in serves as a double safeguard, while a contact diary helps to inform those not using an app, thereby increasing the app’s overall effectiveness.
- Reduce uncertainty and provide users with a clear picture of what will happen in case of infection. Succinctly and clearly describe what exactly the app will do if the user comes in contact with a person who is infected and how infected users can inform others.
- Adhere to current design trends to make the app visually appealing to as many as possible. Allow customized layouts or consider different graphic layouts for different target groups—like younger children, teenagers, and older adults—to better meet their needs and expectations. Prefer the currently widely employed tab navigation, hamburger menu, and card-based layout.
- Address the need of autonomy by providing users with full information and control over data. Settings should be customizable in both directions, allowing users to either pause and delete or share more detailed personal data than currently required.

Recommendations based on the reviewed best practice examples are consistent with other studies on features designed to increase user engagement with mobile health apps [36], especially those providing diary or note-taking functionality, health monitoring features, personalized feedback, personalized information matched to user characteristics (like their region of residence), providing autonomy through customizable settings, and screen design and navigation. Besides inspiration from existing apps and features, recommendations for further development could be driven by either the literature [36-38] or upcoming challenges over the course of the pandemic. First, rewards should be used more intensively (eg, for opening the app, sharing a positive test result with others, or recommending the app to others). Second, notifications and reminders on, for example, updated information or keeping a diary are likely to increase engagement with the app. Third, the perception of personal benefits is a facilitator of contact tracing and may be achieved by providing feedback on what the app has done for the user. Likewise, addressing collective responsibility is likely to facilitate engagement, for example, by providing feedback and rewards on what the user has done for the community and what the community has contributed to pandemic control, or by setting collective goals, for example, pertaining to the number of app recommendations or downloaded apps. Fourth, rewards and incentives could also be linked to the national health policy (eg, access to free of charge testing could be offered to users that received an alert or obtained critical results from a symptom checker). Likewise, using a venue check-in could be rewarded by easier access to social events. Such rewarding *health policy–related app characteristics* seem to have the potential to further increase app adoption. However, those health policy–related characteristics should be developed and implemented in close collaboration with lay publics such as civil society representatives, advocacy groups, and nongovernmental organizations [39]. Fifth, as greater interactivity and personalized features of tracing apps require more personal information (eg, information on regional lockdown rules require information on the user’s location), it is even more important to implement the highest standards of transparency, data privacy, and control over personal data. Nevertheless, implementing those features seems important, as a recent study by Meier and colleagues [40] found that the perceived benefits of tracing apps are more important for the intention to use an app than privacy concerns from a user perspective.

As the percentage of people who have been vaccinated increases, two strategies are possible: tracing app promotion can be slowed or their functionality adapted to the changing situation. The latter should be considered an adaptive strategy, capitalizing on the observation that millions have downloaded the app and the premise that digital solutions to upcoming challenges could be useful. Such an adaptive strategy consists of slowly stepping down tracing features while stepping up the number of features designed to manage the pandemic’s next phase, starting with the option of informing others if they have had contact with a vaccinated and, hence, potentially less infectious person [41] or one who has not yet been vaccinated. The adaptive strategy could include features designed to increase the rate of vaccinations, shifting the target behavior from tracing to undergoing vaccination. It might provide useful and personalized information; support users making appointments for health assessments, treatment, or vaccinations; report adverse or long-term effects; or aid users wanting to contact health care professionals. Designing adaptive tracing apps that are capable of changing their focus depending on a pandemic’s life cycle appears feasible, of benefit to protecting public health, and worthy of further investigation.

### Study Strengths

First, to evaluate quality characteristics, we systematically used an interdisciplinary approach by gathering a team of experts providing both digital health and human–computer interaction perspectives. All the apps were evaluated by four independent raters with two experts from digital health science and two from human–computer interaction research. Second, we included national apps from countries with similar identical legal regulations on voluntariness, privacy, and data protection. By the time this study was conducted, all countries adhered to the European GDPR or equivalent regulations. Therefore, confounding factors related to voluntariness, privacy, and data protection standards are unlikely to affect results to a greater extent. Nevertheless, differences in the integration of tracing apps into the respective health care system and national strategy to fight the pandemic exist.

Third, we investigated associations between app quality characteristics, actual national adoption rates, and infection rates. For the first time, results indicated a direct association between app quality characteristics and adoption rates that were, in turn, associated with lower infection rates. Although causality cannot be inferred from observational data, these results are consistent with prior simulation studies. Unless contradictory experimental data become available, our results should encourage app developers and policy makers to focus more on developing engagement-friendly designs for COVID-19 contact tracing apps to potentially enhance pandemic control. Third, prior reviews investigated all kinds of COVID-19–related apps, including apps with nontracing functionality (eg, only providing information to patients or health care professionals) and apps developed by both governmental and nongovernmental (eg, private technology) agencies [18,34,35]. This study focused entirely on COVID-19 contact tracing apps released by national health authorities, rendering the results especially important to public health officials and policy makers. Fourth, this study provides an estimation on potentially achievable adoption rates in case of optimal app design. For an optimistic outlook, the upper bound of the BCAR 80% highest density interval suggested an adoption rate of 61% for maximum MARS total scores and 54% for maximum engagement scores. However, these estimates also point to the necessity of additional measures beyond app-based digital contact tracing, as adoption rates are still below the thresholds indicated by simulation studies. Especially for younger children or older adults (eg, those with dementia), wearables may be an option to increase the overall adoption of digital contract tracing in the population [2].

### Study Limitations

Several study limitations must be considered. First, app development is an ongoing and dynamic process. Consequently, this review merely provides a snapshot of the current status of app quality. The study is not a final report investigating the success or failure of specific national apps or digital contact tracing in general. Instead, it aims to support developers and public health policy makers striving to improve COVID-19 contact tracing apps, focusing on a predetermined set of important, yet limited, app quality characteristics evaluated by the MARS. Second, the number of downloaded apps was used as our measure of app adoption, and more precise measures exist, like the number of times a user opens the app, how long an app is used, and how many positive test results are shared. Moreover, we do not know the percentage of users who uninstalled the app. In one Swiss study, the uninstallation rate was 5% [29]. On the other hand, more precise measures conflict with data privacy, and the number of downloads was the only measure that was widely available to us. Third, item 15 of the MARS indicates that the correctness of information and scores might be biased toward more positive ratings. In cases of uncertainty, we assumed higher values, mainly because all the apps were released by credible sources: national health authorities. From a technical perspective, we were unable to determine if sensors worked properly and, therefore, if the information on contacts was reliable and accurate. From a medical perspective, COVID-19–related information was not reviewed by an independent board of virologists. However, we are not aware of any leading virologists who have criticized the information provided by any of the tracing apps we reviewed. Likewise, we had no information on diagnostic accuracy (ie, the sensitivity, specificity, and positive and negative predictive values of personal risk evaluations or symptom checkers). App developers should strive to include validated tools, since one study by Munsch and colleagues [42] found that some symptom checkers performed no better than random guessing, while some were highly sensitive, others highly specific, and only two both adequately sensitive and specific. Fourth, MARS, the instrument used in this study, does not include a section on privacy and security, a topic that was widely discussed in the media in the early phases of tracing app development [43]. Vokinger and colleagues [44] recently provided a framework for evaluating this issue in COVID-19 tracing apps.

Fifth, we focused exclusively on the characteristics of tracing apps and were unable to assess how these apps were embedded in the overall health care system or whether there were changes over time in the way tracing apps were embedded. Finally, we only assessed the international English version of each app and did not rule out the possibility that app versions in the language of the app’s country of origin might include more information or features.

### Conclusions

The member states of the European Union, associated countries, and former members with almost identical legal regulations on the voluntariness of use and data protection regulations serve as an excellent real-life laboratory for investigating tracing apps that have been released by the various national health authorities. All 21 national COVID-19 contact tracing apps that we evaluated demonstrated high levels of functionality, aesthetics, and information quality. Although, adoption rates were below the desired levels recommended by several simulation studies investigating the impact of digital contract tracing, while other research indicates that every additional percent app adoption is important. Our results suggest that engagement-friendly app design has the potential to gain those additional percentages. We found a lower level of an engagement-friendly app design, with substantial variations between the various apps. Moreover, app designs that raters considered more engagement-oriented were generally linked to higher app adoption rates, which makes developing more engagement-friendly app designs a promising target as countries strive to optimize their COVID-19 contact tracing apps. Several specific recommendations based on best practice examples were provided. The association we observed between higher app adoption rates and lower infection rates are consistent with predictions from simulation studies and—despite limitations—could indicate that COVID-19 contact tracing apps could contribute to flattening COVID-19 curves.

## Appendix

Multimedia Appendix 1 Overview on COVID-19 contact tracing apps considered for Mobile App Rating Scale rating.

## Abbreviations

BCAR: best-case adoption rate
GDPR: General Data Protection Regulation
ICC: intraclass correlation coefficient
MARS: Mobile App Rating Scale
OLS: ordinary least squares
QR: Quick Response
