# Supplementary material for: Quality and Adoption of COVID-19 Tracing Apps and Recommendations for Development: Systematic Interdisciplinary Review of European Apps
Source: J Med Internet Res. 2021 Jun 2;23(6):e27989. doi: 10.2196/27989 (PMC8174558; doi:10.2196/27989)
Supplement: Multimedia Appendix 1 [file jmir_v23i6e27989_app1.pdf]

Multimedia Appendix 1. Overview on COVID-19 contact tracing applications considered for MARS rating.

Country	Numbers of Inhabitants	App Name	Date of App Release	Downloads	Date of retrieval of Download Numbers	Adoption rate in %	Cumulated Infections until Date of Downloads	Cumulated Infections from App Release until Date of Downloads	Version tested	Releasing Institution
Austria	8,858,775	Stopp Corona	03/25/2020	1,312,063 [a]	12/07/2020	14.81	305,673	300,085	2.0.5 (Android) / 2.0.7 (iOS)	Österreichisches Rotes Kreuz
Belgium	11,455,519	Coronalert	09/30/2020	2,200,000 [b]	12/07/2020	19.20	592,615	474,163	1.8.2 (Android) / 1.2.1 (iOS)	Sciensano - Belgium
Croatia	4,076,246	Stop COVID-19	06/17/2020	78,534 [a]	12/07/2020	1.93	152,239	149,981	2.0.0 (Android)/ 2.0.0 (iOS)	Republika Hrvatska Ministarstvo zdravstva
Czech Republic	10,649,800	eRouška	04/10/2020	1,443,691[a]	12/10/2020	13.56	563,333	557,601	2.1.613 (Android)/ 2.1.4 (iOS)	Ministerstvo zdravotnictví České republiky
England/ Wales	56,602,840	NHS COVID-19	09/24/2020	20,361,253 [b]	12/02/2020	35.97	1,511,028	1,112,787	3.9 (Android) / 3.9 (iOS)	Department of Health & Social Care
Estonia	1,324,820	HOIA	08/20/2020	224,833 [a]	12/09/2020	16.97	16,054	13,827	1.0.4(Android) / 1.3.3 (iOS)	TEHIK
Finland	5,517,919	Koronavilkku	08/31/2020	2,800,000 [a]	12/06/2020	50.74	27,631	19,545	1.2.1 (Android) / 1.2.0 (iOS)	Terveyden ja hyvinvoinnin laitos
France	67,012,883	TousAntiCovid	06/02/2020	11,600,000 [b]	12/18/2020	17.31	2,500,000	2,309,265	2.0.1 (Android) / 2.1.0 to 2.1.8 (iOS)	Gouvernement Francais
Germany	83,019,213	Corona-Warn-App	06/16/2020	24,200,000 [a]	12/18/2020	29.15	1,454,009	1,265,757	1.5.1 (Android) / 1.5.2 to 1.71 (iOS)	Robert Koch-Institut
Iceland	356,991	Rakning C-19	04/01/2020	142,796 [b]	10/27/2020	40.00	4,574	3,354	2.1.2 (Android) / 2.1.2. (iOS)	Embætti landlæknis
Ireland	4,904,240	COVID Tracker Ireland	07/07/2020	2,200,000 [b]	12/01/2020	44.86	72,798	47,260	1.0.4 (Android) / 1.0.4 (iOS)	Health Service Executive (HSE)
Italy	60,359,546	Immuni	06/02/2020	10,000,000 [b]	12/11/2020	16.57	1,806,000	1,572,485	2.1.0 (Android) / 2.1.0 (iOS)	Ministero della Salute
Latvia	1,919,968	Apturi COVID	05/29/2020	263,848 [a]	12/04/2020	13.74	73,948	72,884	1.0 (Android) / 1.3.9 (iOS)	Slimibu Profilakses un Kontroles Centrs
Malta	493,559	CovidAlert Malta	09/18/2020	84,210 [b]	11/20/2020	17.06	8,681	6,047	1.2.8 (Android) / 1.0 (iOS)	Government of Malta
Netherlands	17,282,163	CoronaMelder NL	08/10/2020	4,321,443 [b]	12/23/2020	25.01	731,902	671,844	1.0.4 (Android) / 1.0.11 (iOS)	Rijksoverheid
Poland	37,972,812	STOP COVID - ProteGO Safe	10/13/2020	1,637,927 [a]	12/10/2020	4.31	1,102,000	966,722	4.6.0 (Android) / 4.6.0 (iOS)	Ministerstwo Cyfryzacji

Country	Numbers of Inhabitants	App Name	Date of App Release	Downloads	Date of retrieval of Download Numbers	Adoption rate in %	Cumulated Infections until Date of Downloads	Cumulated Infections from App Release until Date of Downloads	Version tested	Releasing Institution
Portugal	10,276,617	STAYAWAY COVID	09/01/2020	2,822,522 [a]	12/16/2020	27.47	358,296	300,053	1.0.5 (Android) / 1.0.5 (iOS)	FCT - Fundação para a Ciência e a Tecnologia I.P.
Scotland	5,463,300	Protect Scotland	09/10/2020	1,700,000 [b]	12/14/2020	31.12	108,537	85,992	1.0.4 (Android) / 1.0.4 (iOS)	NHS Education for Scotland
Slovenia	2,080,908	#OstaniZdrav	08/18/2020	297,000 [b]	12/17/2020	14.27	102,043	99,587	1.3.1 (Android) / 1.3.2 (iOS)	National Institute of Public Health
Spain	46,937,060	Radar COVID	07/07/2020	6,571,600 [b]	11/27/2020	14.00	1,628,000	1,602,787	1.0.7 (Android) / 1.0.8 (iOS)	Ministerio de Asuntos Económicos y Transf. Digital
Switzerland	8,544,527	SwissCovid	06/25/2020	2,835,331 [a]	12/07/2020	33.18	354,306	322,878	1.1.2 (Android) / 1.1.2 (iOS)	Federal Office of Public Health

Note: Numbers of inhabitants are drawn from Eurostat (2019) and Gov UK (2019).

[a] Statistics on downloads from app developers

[b] Downloads retrieved from online resources
